# How Do People Cope During the COVID-19 Pandemic and Stay Well? A Salutogenic Longitudinal Study in Israel

**DOI:** 10.3389/fpsyg.2021.729543

**Published:** 2021-10-26

**Authors:** Adi Mana, Or Catz, Yossi Mana, Moran Neuman, Sharon Benheim, Shifra Sagy

**Affiliations:** ^1^Department of Behavioral Studies, Peres Academic Center, Rehovot, Israel; ^2^Department of Psychology, Ashkelon Academic College, Ashkelon, Israel; ^3^Martin Springer Center for Conflict Studies, Ben-Gurion University of the Negev, Beersheba, Israel

**Keywords:** sense of coherence (SOC), longitudinal study, COVID-19, social support, well-being, mental health, sense of national coherence (SONC), anxiety

## Abstract

Employing the salutogenic approach, this longitudinal study explored the effects of coping with the COVID-19 pandemic in Israel, as it evolved from an acute to a chronic stress situation, during the first year. We examined the role of individual [sense of coherence (SOC)], social (perceived social support), and national [sense of national coherence (SONC)] coping resources, as well as situational and demographic factors, in predicting mental health and anxiety. Data was collected in five phases between March 2020 and February 2021 via a repeated panel sample and included 198 Jewish Israelis (52% males) age 18–64 (*M* = 43.5). The results confirmed the expected pattern of moving from acute to chronic stressful situation: levels of general anxiety were higher in the first phase of the pandemic outbreak as compared to the other phases. Levels of social and national coping resources significantly decreased over time. However, as expected, the salutogenic resource of the individual sense of coherence remained stable and was also found as the main predictor of both anxiety and mental health in the 5 phases of the study. Beyond the explanatory factor of SOC, mental health was better explained by the social and national coping resources, while anxiety was explained by situational factors (level of financial risk and gender). The discussion delineates the longitudinal effects of individual, social, and national coping resources on mental health and anxiety during the dynamic process of the long period of 1 year of the pandemic, evolving from acute to chronic phases of the complicated health, economic, social, and political crisis

## Introduction

The COVID-19 pandemic evolved from an acute and sudden stressful crisis among populations all around the world into a long period of struggling with the virus in daily life and with its consequences. The spread of the virus over several waves has demanded continuing efforts on individual, social, and national levels to find ways to live with the pandemic. The pandemic pattern has been characterized by waves of fast-growing numbers of confirmed COVID-19 cases, increase in death rate, feelings of losing control, and implementation of rigid restrictions that slowly eased until the next wave began rising (Roser et al., 2020). The current study examined the long-term effects of “living with the COVID-19 pandemic waves” and the coping patterns over time during the first year of the crisis in Israel.

### The Salutogenic Approach

Most of the research regarding coping with the COVID-19 pandemic asks pathogenic questions and describes the negative effects of the crisis on well-being, anxiety, and depression (for reviews see Salari et al., 2020; Prati and Mancini, 2021). Our study aims to explore the longitudinal effects of the pandemic on mental health and anxiety levels among the population in Israel during the first year of the pandemic, employing the salutogenic theoretical approach (Antonovsky, 1979). This approach is mainly focused on the question “Why, when people are exposed to the same stress which causes some to become ill, do some remain healthy?” (Antonovsky, 1979, p. 56) and suggested using a different perspective than the pathogenic one by focusing on individual and collective resources and positive outcomes of challenge and crisis.

However, Antonovsky (1987) did not call for a complete shift away from pathogenesis, but rather a shift toward salutogenesis alongside pathogenesis and conceptualizing health along a continuum from ease to dis-ease. This approach leads us to ask not only about the process that leads to both salutogenic and pathogenic results (e.g., mental health and anxiety), but also to focus on the role of coping resources during a longitudinal crisis. Thus, our study examined the longitudinal role of individual (sense of coherence), social (perceived social support), and national (sense of national coherence) coping resources, as well as situational and demographic factors (level of health and financial risk, gender, and marital status) in predicting mental health and anxiety at different time points during the COVID-19 crisis.

### Mental Health and General Anxiety During the COVID-19 Pandemic

Mental health is defined as a salutogenic factor related to general coping resources. According the World Health Organization (WHO), the definition of mental health is “*a state of well-being in which the individual realizes his or her abilities, can cope with the normal stresses of life, can work productively and fruitfully, and is able to make a contribution to his or her community*” (World Health Organization, 1998). According to this definition, mental health includes the presence of positive feelings, positive functioning in individual life and community life, emotional and psychological life satisfaction, and refers to how people evaluate their lives. We explored mental health using the MHC-SF that tests emotional, psychological, and social well-being (Lamers et al., 2011). Previous results indicated that the MHC-SF is highly reliable over time (Lamers et al., 2011). Studies conducted during COVID-19 revealed that levels of mental health and well-being were strongly related to coping resources of SOC (Barni et al., 2020; Schäfer et al., 2020), trust in authorities (Falcone et al., 2020; Généreux et al., 2020) and social support (Saltzman et al., 2020; Szkody et al., 2020). However, a review of recent studies cautiously indicated that due to the fluctuating nature of the pandemic and the restrictions of COVID-19 regulations, coping resources have become progressively limited as the pandemic continued (Danioni et al., 2021; Savolainen et al., 2021). For example, social distancing and lockdown regulations which limited daily life’s social interactions increased feelings of loneliness and decreased social support coping resources (Saltzman et al., 2020). In the national sphere, conspiracy theories, anger, and resistance to national regulations led, in many countries, to decreased levels of public trust in the authorities who attempted to manage the pandemic (Almutairi et al., 2020; Marinthe et al., 2020).

Therefore, we predicted that level of mental health, at different phases of the pandemic, would be explained by the coping resources available to the individual (at the individual, social, and national levels). Moreover, we expected that as long as the crisis has been prolonged and become chronic, the mental health levels would decrease, since the availability of coping resources would decrease.

While mental health is considered a salutogenic response to the prolonged crisis, general anxiety is a pathogenic symptom of stressful situations and usually considered the main response to situations of acute stress, mainly affected by the nature of the situation (Grillon et al., 2007). The first sudden spread of the COVID-19 virus could be described as an acute life event which required major behavioral adjustments within a relatively short period of time (Boyraz and Legros, 2020; Prati and Mancini, 2021). The pandemic then gradually evolved into a chronic stressor with persistent or recurrent demands which required adjustments over prolonged periods of time (Patel and Raphael, 2020; Qi et al., 2021). Since acute stress response shifts the physiological system to a state of higher vigilance and physical capacity (“fight or flight”), the intensity of the reactions during the acute phase is mainly influenced by the overwhelming nature of the situation itself and the perceived level of risk (Kudielka and Wüst, 2010; Bareket-Bojmel et al., 2020). Prolonged exposure to stressful events, on the other hand, were found to overwhelm and decrease peoples’ physical, psychological, and social coping resources, reduce individuals’ abilities to cope or readjust to chronic stressors, and increase the probability that psychological distress or disorder will follow (Lazarus and Folkman, 1984; Tennant, 2002).

Although acute and chronic stress are considered distinct types of stress, with different effects on stress responses and coping resources, there is some ambiguity and difficulty determining whether stressful experiences are chronic or acute in nature (Gottlieb, 1997). Some reasons for this could be the interaction between acute stressors and former chronic stressors, transmission of distress from one life sphere to another, coping responses that are employed to moderate the stressors’ impact, and fluctuation in levels of stress through a time period (Gottlieb, 1997; Sagy, 2002). Therefore, we have not distinguished between two dichotomic stress situations: acute vs. chronic stress, but rather examine longitudinal effects of coping with COVID-19 pandemic that reflect the process of moving from an acute stress situation (the sudden outbreak of the first wave of the pandemic) throughout significant times of change in the pandemic during the first year, when the stressful situation gradually became chronic.

A growing body of research was conducted in the acute phase of the COVID-19 pandemic (Breslau et al., 2021; Prati and Mancini, 2021; Qi et al., 2021). Longitudinal studies which have been conducted in the acute phase of the pandemic and were compared to pre- or post-lockdown measures (Breslau et al., 2021; Gallagher et al., 2021; Prati and Mancini, 2021) showed an increase in the levels of anxiety, stress, loneliness, and depression during the outbreak of the pandemic. Therefore, we expected that at the acute phase of the overwhelming outbreak of the “mysterious virus” and the extraordinary experience of global lockdowns, the pathogenic outcome of anxiety response would be high, and then would gradually decrease over the following phases, as the understanding of the virus and ways to manage became clearer. Moreover, we expected that the situational factors and perceived level of risk, would predict the levels of general anxiety.

### Coping Resources on the Individual Level: Sense of Coherence

Antonovsky (1979) claimed that sense of coherence is the key to understanding “Why, when people are exposed to the same stress which causes some to become ill, do some remain healthy?” (Antonovsky, 1979, p. 56). Sense of coherence (SOC) suggested as a core coping resource and defined as a “global orientation that expresses the extent to which one has a pervasive, enduring though dynamic feeling of confidence” (Antonovsky, 1987, p. 19). SOC expresses an individual view of the world as comprehensible (the extent to which stimuli from one’s external and internal environment are perceived as structured, explicable, and predictable), manageable (the extent to which resources are perceived as available to a person to meet the demands posed by these stimuli), and meaningful (the extent to which these demands are perceived as challenges, worthy of investment, and engagement). According to the Salutogenic approach, SOC became a stable coping resource during adulthood, and helps to identify and mobilize relevant resources to cope with stressors and manage tension successfully (Antonovsky, 1987), such that it promotes health and well-being. The salutogenic hypotheses have been explored over the last four decades and studies have confirmed the important role of SOC in predicting and explaining health in regular life as well as in crisis or disaster times (for review, see Eriksson and Mittelmark, 2017).

Recent studies highlight the important role of SOC in adjustment to the pandemic. For example, Schäfer et al. (2020) explored the longitudinal effects of SOC on level of mental health and pointed out that SOC predicted changes in psychopathological symptoms from COVID-19 pre-outbreak (at the end of February) to post-outbreak (1 month later). The results showed that higher levels of SOC buffered the impact of COVID-19 stressors on general health but did not result in lower symptom levels.

International comparative studies revealed the unique contribution of SOC to reduction of anxiety and strengthening of mental health in the early phases of the pandemic (Barni et al., 2020; Généreux et al., 2020; Schäfer et al., 2020). Mana et al. (2021b) explored the role of the coping resources of SOC and other social and national coping resources and risk factors, in predicting mental health and anxiety among participants in four countries (Israel, Italy, Spain, and Netherlands) during the first months of the COVID-19 pandemic. The results revealed that SOC was found to be the main predictor of both anxiety and mental health. However, the situational factor of level of financial risk was a better predictor of anxiety, while SOC and other coping resources were more dominant in explaining mental health.

An additional study explored “how does SOC work” during the COVID-19 pandemic and tested the salutogenic assumption that a strong SOC allows one to reach out in any given situation and find those resources appropriate to the specific stressor (Mana et al., 2021a). Data collected among participants from seven countries confirmed the suggested mediation model and revealed that perceived family support and trust mediated the relationships between SOC and mental health, controlling for gender, level of exposure to the virus, and level of health and financial risk (Mana et al., 2021a).

Based on these studies, we expected that SOC, as a core and constant coping resource, would remain stable and would have a major role in promoting mental health and reducing levels of anxiety during the different phases of data collection.

### Coping Resource on the National Level: Sense of National Coherence

Levine and Scotch (1970) claimed that “Stress is not an individual affair but must be viewed in terms of the social context in which it occurs” (p. 287).

Since the COVID-19 global pandemic has become an international crisis in which each nation led its own way Žižek (2020), the current study explored the role of sense of national coherence (SONC) as another core coping resource. SONC, based on Antonovsky’s components of SOC, is defined as an enduring tendency to perceive the national group as comprehensible, meaningful, and manageable (Sagy, 2014; Mana et al., 2019). SONC is different from SOC, which is a core and stable coping resource: SONC is related to the perceptions of one’s nation, and therefore is more sensitive to political situations and intergroup relationships. This assumption was tested in several research conducted among participants in different contexts of intergroup conflict (for review, see Sagy and Mana, in press). Consistent relationships were found between levels of SONC and openness to the others’ narratives. SONC was strongly related to the tendency to adhere to the ingroup narratives and reject the outgroup narratives (Mana et al., 2019).

Sense of national coherence was found to be an important coping resource contributing to mental health during the COVID-19 pandemic and correlated with trust in governmental institutions responsible for managing the pandemic in data from four countries collected during the acute phase of the pandemic (Mana et al., 2021b). However, stronger levels of SONC were found among right wing voters as compared to left wing voters in Israel and in the United States (Mana and Sagy, 2020; Hardy et al., 2021).

Thus, we expected that perception of the national group as a source of a high level of SONC would predict mental health and anxiety. However, we expected that it would decrease over the 5 phases of data collection, while the national, social, and political crisis would deepened.

### Coping Resources on the Social Level: Perceived Social Support

Another important resource well documented in stress literature is *Perceived Social Support* (Zimet et al., 1988), which is a subjective feeling of being supported and cared for by others and having a reliable network to turn to when needed, in everyday situations or specific moments of crisis. This can be perceived as coming from different sources, e.g., family, significant others, and/or community (Zimet et al., 1988). People with access to supportive social relationships were found to show better health, and success in coping better with stressful events (for review, see Taylor, 2011). Several studies have revealed the important role of social support on mental health and stress during the COVID-19 pandemic (e.g., Kimhi et al., 2020; Szkody et al., 2020). Research from different social contexts found that as a result of social distancing regulations levels of social support decreased, and feelings of isolation and loneliness increased (Généreux et al., 2020; Saltzman et al., 2020; Szkody et al., 2020). Therefore, we expected that perceived social support would promote mental health and reduce level of anxiety but would also be less available to people and diminished over time during the pandemic.

### Situational Factors

The COVID-19 pandemic involved different risk factors: mainly health and economic risk (Bareket-Bojmel et al., 2020). Evidence revealed the negative impacts on mental health of unemployment and financial stress as well as stress and worries about the health of oneself or others, which people experienced early in the pandemic (Boyraz and Legros, 2020). Bareket-Bojmel et al. (2020) compared three countries (the United States, the United Kingdom, and Israel) during the COVID-19 outbreak and explored the relationships between four sources of anxiety: health-related, economic-related, change in daily routine, and anxiety generated by social isolation. Results show that in all three countries levels of economic and health anxiety were essentially equal, and both surpassed routine-change and isolation anxiety. However, since the financial crisis increased as the pandemic continued, especially among vulnerable populations (Roser et al., 2020), we expect that participants who estimated that due to the pandemic they would face financial difficulties, would also have high levels of anxiety. In addition to financial and health risks, several studies found that vulnerable groups tended to be more negatively affected by the COVID-19 crisis. Gender was found as a crucial risk factor and related to higher levels of anxiety in different countries (e.g., Mana et al., 2021b), especially during the first month of the pandemic outbreak (Breslau et al., 2021). However, the relationship between marital status and levels of psychological distress is not clear. The vulnerability of people living alone seemed to increase due to the regulations of social distancing, quarantine, and lockdown, while married individuals coped better, were less stressed, and reported higher self-esteem (Lawal et al., 2020). However, the effect of marital status was not found in other studies (e.g., Lawal et al., 2020).

### COVID-19 in Israel

Similar to other countries, the pandemic in Israel was not only a health crisis and has resulted in causing or deepening social, economic, political, and national crises (Žižek, 2020). In Israel the onset of the pandemic occurred in the midst of an existing and prolonged political crisis, after a third round of elections that led to growing gaps and expressions of hatred between the political camps as the repeated elections failed to achieve a viable government. The struggle to control the pandemic in Israel became a main factor in the Israeli politics in the first year of the pandemic: it was presented as the main reason to compromise and create a “unity” government, which influenced the level of trust in governmental institutions responsible for controlling the pandemic and deepened the gap between political camps and between social groups in Israel (Mana and Sagy, 2020; Louwerse et al., 2021).

Braun-Lewensohn et al. (2021) explored the relationships between SOC, hope, risk factors, and levels of psychological distress during the first and second lockdowns as compared to non-crisis times among members of different social groups in Israel. These findings revealed increased levels of distress while the main contribution to protection of this distress was SOC. Another longitudinal study (Kimhi et al., 2020) compared sense of danger, distress symptoms, and national resilience during the peak of the first wave of COVID-19 with data collected 2 months later: sense of danger, distress symptoms, and national resilience significantly decreased, while perceived well-being increased.

While these studies reflect the major impact of the COVID-19 crisis on the Israeli population during the beginning of the pandemic, the current study asked about the differential effects and patterns of coping during 5 phases of the crisis, from the initial acute to the later chronic phase.

Research hypotheses:

(1) We expected the following relationships between the coping resources, the situational factors, and the salutogenic and pathogenic outcomes:
(a) Levels of mental health will be predicted by higher levels of coping resources (SOC, perceived social support, and SONC) and lower levels of financial risk and health risk.
(b) Levels of anxiety will be predicted by lower levels of coping resources (SOC, perceived social support and SONC) and higher levels of financial risk and health risk.
(c) Based on the salutogenic approach, we expected SOC to be the main predictor of both salutogenic and pathogenic outcomes (mental health and anxiety).
(d) Based on former studies, we expected that the salutogenic measure of mental health would be better explained by the social and national coping resources, while anxiety, the pathogenic measure, would be explained more by situational factors (gender, level of health and financial risk).
(2) We expected to find changes between the different phases of data collection:
(a) Based on studies related to the differences between acute and chronic stress we expected fluctuations in levels of anxiety: during the acute state of the initial outbreak of COVID-19 we expected higher levels of anxiety with a gradual decrease over time as the crisis continued (phases 2–5).
(b) Based on previous studies related to the decreased level of mental health during chronic stress, we expected to find a continuing decrease in mental health over the 5 phases of data collection.
(c) Based on previous research related to the decreased level of coping resources during chronic stress, we expected to find a continuing decrease in coping resources of perceived social support and SONC over the 5 phases of data collection.
(d) Based on the Salutogenic approach, we expected SOC, as a core and constant coping resource, to be stable across the phases.
(e) Based on accumulating data related the COVID-19 financial crisis in Israel, we expected financial risk to increase as long as the crisis continued.

## Materials and Methods

### Participants

**FIGURE 1:**
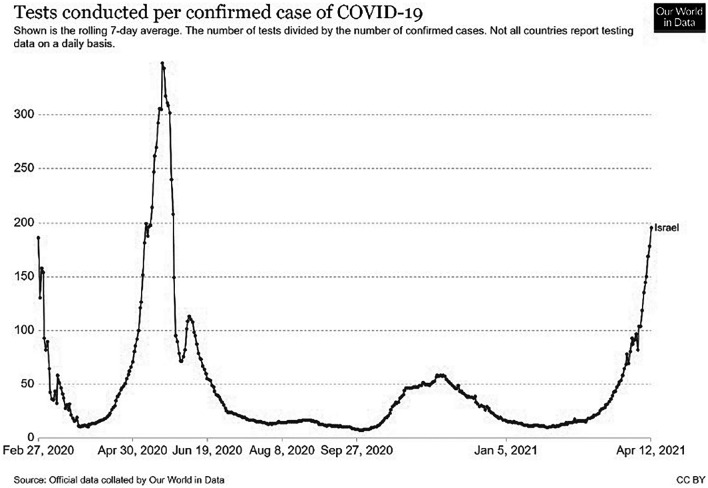
Test conducted per confirmed cases of COVID-19 in Israel.

Data collection took place between March 2020 and February 2021 (see Figure 1 for situational data in each phase). Participants were recruited by the Midgam Project Web Panel (Midgam Panel, 2020). The panel is a non-probability, general population panel and uses a stratified sampling method (Callegaro et al., 2014).

During registration, participants provided information that prevented double registration and enabled a stratified quota sampling based on known demographic distribution of the population. The participants received money for their participation.

The data was collected from among sample of 369 Jewish Israelis. The current study analyses were conducted on the results of 198 participants who completed the questionnaire in all five phases of data collection. Chi-square tests between the longitudinal study sample and the total sample revealed no significant differences in age, education, marital status, financial, and health risk. The current sample included (103 males, 52.0%), with age range of 18–64 years (*M* = 43.5, SD = 12.2). About 30% had high school education or less, about 30% had vocational education, and about 30% had completed a BA degree. As shown in Table 1, most of the participants were married and secular. The number of children they reported ranged between 0 and 10 (*M* = 2.01, SD = 1.9). Very few participants in each phase reported that they had been or were diagnosed with Coronavirus [0 (0.0%), 1 (0.5%), 6 (3.0%), 15 (7.6%), 17, (8.6%), respectively]. About a quarter (23.2%) of the participants reported that they were in a high-risk group because of their age or health status.

**TABLE 1:** Description of the gender, marital status, religious, education, and risk group variables.

Variables	Categories	*n*	%
Gender	Male	103	52.0%
	Female	95	48.0%
Marital status	Single	51	25.8%
	Married	132	66.7%
	Divorced	14	7.1%
	Widow	1	0.4%
Religious	Secular	104	52.5%
	Traditional	41	20.7%
	Orthodox	34	17.2%
	Ultra orthodox	19	9.6%
Education	High school or less	56	28.3%
	Vocational education	62	31.3%
	BA students or graduate	53	26.8%
	MA students or higher	27	13.6%
Risk group	Yes	46	23.2%
	No	152	76.8%

We conducted power analysis using G^∗^power (Faul et al., 2007) for the repeated measures ANOVA and for the regressions based on medium effect size and power >95% with *a priori* alpha set at 0.05. Power analysis for Linear multiple regression showed that 160 participants were needed and power analysis for repeated measures ANOVA showed that 65 participants were needed.

### Instruments

The study instrument comprised structured and self-reported questionnaires.

*Mental Health Continuum (MHC-SF, Lamers et al., 2011)* This scale includes 14-items measuring the three components of well-being: emotional, social, and psychological. The questionnaire was adapted to the current context and based on the experiences the participants had over the last 2 weeks (*never*, *once in these 2 weeks, about once a week*, *two or three times a week*, *almost every day*, or *every day*). Internal consistency of the questionnaire was estimated at 0.89 (Lamers et al., 2011) and in the current study α were 0.92, 0.92, 0.92, 0.93, and 0.93 (each phase separately).

*Sense of Coherence (SOC-13, Antonovsky, 1987)* The SOC measure includes 13 items, on a 7-point Likert scale, which explore the participants’ perceptions of the world as comprehensible, meaningful, and manageable. The α values of the SOC-13 versions range from 0.70 to 0.92 (see Eriksson and Mittelmark, 2017) and in this study the α were 0.81, 0.83, 0.85, 0.83, and 0.85 (each phase separately).

*Sense of National Coherence (SONC, Mana et al., 2019)* The eight items on a 7-point Likert scale (1 = totally agree, 7 = totally disagree) explore the participants’ perceptions of his/her own society as comprehensible, meaningful, and manageable. Internal consistency of the questionnaire was estimated at 0.80 (Mana et al., 2019) and in the current study α = 0.86, 0.87, 0.86, 0.87, and 0.89 (each phase separately).

#### Perceived Social Support

Five items explored feelings of support that the participant feels he/she receives from social circles that were found in previous studies as relevant for coping with stress (Zimet et al., 1988). The participants were asked “How often, over the last 2 weeks, have you felt support in each of these social circles” (family, friends, community in the neighborhood or settlement, virtual communities such as social networks, Twitter, Facebook, and workplace) on a 5-point Likert scale (1 = very much, 5 = not at all) (Internal consistency in the current study were α = 0.72, 0.77, 0.75, 0.75, 0.74 (each phase separately).

#### Level of Exposure to COVID-19 (Financial and Health Risk Factors)

We explored exposure to both health and financial risks due to the COVID-19 crisis by asking if the participant: (1) Had been diagnosed with COVID-19 (Yes/No); (2) Had a close family member who had been diagnosed with COVID-19 (Yes/No); (3) Do you belong to a health risk group due to your age or health condition (yes or no); and (4) To what extent do you think you will suffer financially from the Corona virus crisis? (1 = not at all, 5 = extremely).

#### Socio-Demographic Variables

Demographic information of gender, age, marital status (single, married, divorced, widow, other), education (high school or less, vocational education, BA student or graduate, MA student or higher),

### Procedure

The current study followed the first year of COVID-19 in Israel. During this year the Israeli society experienced three waves of increased numbers of positive cases of COVID-19 and three lockdowns (see Figure 1). Our aim was to explore how people ‘‘live with COVID-19 waves,’’ therefore we tried to collect the data during main points of fluctuation and changes in the nature of the pandemic. Since this period was influenced by many changes it was not possible to predict the specific time point of each change, so we tried to capture the increase and decline of the waves by following the increased and decreased number of positive cases of the virus and the lockdowns that aimed at controlling them^1^. Data collection was conducted along 5 phases of the crisis: starting from phase 1 (25th–27th March, 2020) – the outbreak of the pandemic and the first lockdown; phase 2 (20th–25th May, 2020) – the decline of the first wave, children returned to schools, regulations were removed gradually, and there was a sense of relief and of overcoming the virus; phase 3 (5th–24th August, 2020) – after a period of almost total reduction of COVID-19 cases, the numbers of positive cases increased again, with lockdown in specific places with high numbers of positive cases; phase 4 (26th November – 23th December, 2020) after the second wave and a long lockdown during the holiday season, there was a gradual opening up of the market, although numbers of positive cases were still rising – at this stage the vaccination project began; and phase 5 – after a third lockdown and the continuation of the successful mass vaccination campaign (at this stage more than two and a half million people in Israel had received the first injection).

Prior to data collection, we obtained approval from the ethic committee of Ben-Gurion University of the Negev. Recruitment of participants was conducted via online survey among participants from a general population panel (Midgam Panel, 2020). And their details were saved in the panel system and not shared with the researchers. This way the anonymity of the participants was guaranteed. No identifying data was collected in the questionnaire.

An invitation letter was sent to panelist according to criterion of religion (Jews), age groups, and gender. The letter explained that the research objective was to understand the participant’s experience during the period of COVID-19 crisis. Researcher’s contact name and email address were provided for more information.

## Results

All the analysis were corrected for multiple comparisons using the Bonferroni-Holm (Holm, 1979) correction.

### Correlations Between the Main Variables

In the first step of data analysis Pearson correlations were conducted to explore the association between the main variables of the research during each phase (see Table 2). Significant negative correlations were found between mental health and anxiety for each phase.

**TABLE 2:** The correlations between main variables of the research, separately for each phase (*n* = 198).

Variables	T	MH	Anxiety	SOC	Support	SONC
Anxiety	1	−0.300***				
	2	−0.395***				
	3	−0.421***				
	4	−0.465***				
	5	−0.377***				
SOC	1	0.574***	−0.545***			
	2	0.577***	−0.544***			
	3	0.563***	−0.581***			
	4	0.652***	−0.602***			
	5	0.592***	−0.517***			
Support	1	0.472***	−0.146	0.341***		
	2	0.614***	−0.153	0.364***		
	3	0.517***	−0.281***	0.424***		
	4	0.515***	−0.276***	0.398***		
	5	0.531***	−0.236***	0.346***		
SONC	1	0.396***	−0.133	0.229***	0.299***	
	2	0.294***	−0.092	0.154	0.387***	
	3	0.336***	−0.186**	0.246***	0.339***	
	4	0.415***	−0.189**	0.186**	0.419***	
	5	0.410***	−0.128	0.203**	0.350***	
Financial risk^1^	1	−0.173	0.219**	−0.163	−0.191**	−0.131
	2	−0.286***	0.266***	−0.251***	−0.232***	−0.153
	3	−0.121	0.201**	−0.136	−0.182*	−0.063
	4	−0.130	0.210**	−0.204**	−0.172*	−0.024
	5	−0.165	0.223**	−0.129	−0.124	−0.080

*p < 0.05, **p < 0.01, ***p < 0.001.

*MH, mental health; SOC, sense of coherence; Support, perceived social support; SONC, sense of national coherence.*

*^1^180 participants answered the question about financial risk, as opposed to 198 participants who completed the questions for the other variables.*

Mental health was significantly correlated with the three coping resources (SOC, perceived social support, and SONC), for each phase. However, it was significantly correlated with financial risk only in phase 2.

As for anxiety levels, significant negative correlations were found between anxiety and SOC, and significant positive correlations were found between anxiety and financial risk, for each time phase. However, significant negative correlations were found between anxiety and perceived social support only in phases 3, 4, and 5 and between anxiety and SONC only in phases 3 and 4 (see Table 2).

### Coping Resources and Risk Factors as Predicting Mental Health and Anxiety Levels

The first hypothesis related to the different pattern of coping resources and risk factors which explained mental health and anxiety. We expected that mental health would be predicted by higher levels of coping resources (SOC, SONC, and perceived social support) and lower levels of health and financial risks, while the opposite pattern would be found as related to anxiety (hypotheses 1a,b).

However, while we expected SOC, as a core coping resource, would be the main predictor of both mental health and anxiety during different phases of the pandemic (hypothesis 1c), we expected the salutogenic factor of mental health would be explained more by social and national coping resources, perceived social support and SONC, while anxiety, as a pathogenic factor, would be explained more by situational factors such as gender and level of health and financial risk (hypothesis 1d).

To explore the hypothesis, separate hierarchic regressions were conducted in each of the 5-research phases, for mental health and anxiety as dependent variables. The variables were stepped into the equation according to the following rational: First, the situational factors of levels of health risk and financial risk due to the COVID-19, and the demographic variables that were found in other studies as risk factors: gender and marital status. Afterward, the coping resources were entered, from the individual level to the broader circles of social and then national coping resources. Therefore, in the first step the situational and demographic risk factors we entered: gender, marital status, health risk group, and financial risk. In the second step the individual coping resource of SOC was entered. In the third step the social coping resource perceived social support was entered. And in the last step, the national coping resource SONC was entered. In phases 2–5, the predicted variable from the previous phase was entered in the first step in order to control for that. For example, when predicting the mental health in the second phase, the mental health of the first phase was entered in the first step of the hierarchic regression.

### Mental Health

As shown in Table 3 the regressions from phase 2 to 5 were very similar to each other. For example, the first, the second, and the third steps were significant. In the first step the previous mental health significantly predicted mental health. In the second step SOC and the previous mental health predicted mental health. In the third step perceived social support, SOC, and the previous mental health predicted mental health. However, in the fourth step SONC did not predict mental health but perceived social support, SOC, and previous mental health did.

**TABLE 3:** Beta values for predicting mental health by coping resources and situational and demographic variables, separately for each phase.

Variables	Phase 1	Phase 2	Phase 3	Phase 4	Phase 5
**Step 1**					
Male	−0.010	−0.039	0.053	−0.001	−0.023
Married	−0.071	0.045	0.015	−0.068	0.008
Risk group	0.012	0.050	−0.006	−0.021	−0.018
Financial risk	−0.167	−0.135	−0.015	−0.027	−0.039
Previous MH		0.667***	0.756***	0.795***	0.784***
*R* ^2^	3.5%	50.6%***	57.5%***	64.4%***	62.6%***
ΔR^2^	3.5%	50.6%***	57.5%***	64.4%***	62.6%***
**Step 2**					
Male	−0.058	−0.056	0.046	−0.017	−0.038
Married	0.020	0.076	0.021	−0.017	0.030
Risk group	−0.007	0.030	−0.034	−0.054	−0.045
Financial risk	−0.085	−0.087	−0.003	0.022	−0.026
Previous MH		0.535***	0.655***	0.632***	0.666***
SOC	0.572***	0.314***	0.175**	0.341***	0.215***
*R* ^2^	34.0%***	57.9%***	59.4%***	72.3%***	65.5%***
ΔR^2^	30.5%***	7.3%***	1.9%**	7.9%***	2.9%***
**Step 3**					
Male	0.003	−0.051	0.046	−0.010	−0.032
Married	0.081	0.112*	0.054	0.003	0.059
Risk group	0.008	0.043	−0.033	−0.067	−0.046
Financial risk	−0.040	−0.055	0.012	0.038	−0.015
Previous MH		0.432***	0.588***	0.584***	0.593***
SOC	0.472***	0.268***	0.159***	0.300***	0.207***
Support	0.322***	0.314***	0.142*	0.182***	0.168***
*R* ^2^	42.3%***	65.5%***	60.6%***	74.8%***	67.6%***
ΔR^2^	8.3%***	7.6%***	1.2%*	2.5%***	2.1%***
**Step 4**					
Male	−0.012	−0.050	0.042	−0.016	−0.039
Married	0.075	0.113	0.054	0.003	0.065
Risk group	0.009	0.042	−0.031	−0.063	−0.041
Financial risk	−0.027	−0.055	0.012	0.034	−0.014
Previous MH		0.434***	0.577***	0.564***	0.562***
SOC	0.444***	0.267***	0.157**	0.305***	0.214***
Support	0.266***	0.317***	0.134*	0.159***	0.153**
SONC	0.217***	−0.010	0.041	0.065	0.084
*R* ^2^	46.5%***	65.5%***	60.7%***	75.1%***	68.2%***
ΔR^2^	4.2%***	0.0%	0.1%	0.3%	0.6%

*p < 0.05, **p < 0.01, ***p < 0.001.

*Male – 1 = male, 0 = female; Married – 1 = married, 0 = not married; Risk group – 1 = yes, 0 = no; SOC, sense of coherence; support, perceived social support; SONC, sense of national coherence.*

In the first phase, when we did not have previous mental health in the regression, the second, the third, and the fourth steps were significant. In the first step none of the variables was significant. In the second step SOC predicted mental health. In the third step perceived social support and SOC predicted mental health. Lastly, in the fourth step SONC, perceived social support, and SOC predicted mental health.

Thus, the stronger the previous mental health, the SOC, and the perceived social support, the higher levels of mental health participants reported each time they completed the questionnaire. Additionally, when the previous mental health was not in the regression (in phase 1), the stronger the SOC was, the higher the levels of mental health were.

### Anxiety

As shown in Table 4 the regressions from phase 2 to 5 were very similar to each other. For example, only the first and the second steps were significant. In the first step financial risk significantly predicted anxiety levels only in phase 2, but previous anxiety predicted anxiety in the regressions from phase 2 to 5. In the second step SOC predicted anxiety levels in the four regressions. Perceived social support and SONC that were entered in the third and the fourth steps, respectively, were not significant at all.

**TABLE 4:** Beta values for predicting anxiety by coping resources and situational and demographic variables, separately for each phase.

Variables	Phase 1	Phase 2	Phase 3	Phase 4	Phase 5
**Step 1**					
Male	−0.295***	0.058	0.054	−0.046	−0.060
Married	−0.087	0.030	−0.053	0.141	−0.086
Risk group	0.027	−0.066	−0.086	−0.034	−0.048
Financial risk	0.210**	0.207***	0.059	0.065	0.096
Previous anxiety		0.613***	0.550***	0.581***	0.660***
*R* ^2^	13.3%***	43.2%***	32.8%***	39.4%***	47.4%***
ΔR^2^	13.3%***	43.2%***	32.8%***	39.4%***	47.4%***
**Step 2**					
Male	−0.250***	0.034	0.031	−0.022	−0.041
Married	−0.171**	−0.019	−0.074	0.079	−0.105
Risk group	0.045	−0.040	−0.019	−0.006	−0.024
Financial risk	0.135	0.146**	0.056	0.029	0.078
Previous anxiety		0.475***	0.347***	0.409***	0.546***
SOC	−0.528***	−0.308***	−0.379***	−0.387***	−0.279***
*R* ^2^	39.3%***	50.1%***	42.6%***	50.2%***	53.5%***
ΔR^2^	26.0%***	6.9%***	9.8%***	10.8%***	6.1%***
**Step 3**					
Male	−0.252***	0.035	0.029	−0.023	−0.044
Married	−0.173**	−0.016	−0.087	0.075	−0.112
Risk group	0.044	−0.039	−0.021	−0.003	−0.025
Financial risk	0.134	0.151**	0.049	0.025	0.074
Previous anxiety		0.474***	0.345***	0.410***	0.541***
SOC	−0.525***	−0.317***	−0.358***	−0.372***	−0.267***
Support	−0.011	0.029	−0.056	−0.042	−0.047
*R* ^2^	39.3%***	50.2%***	42.8%***	50.4%***	53.6%***
ΔR^2^	0.0%	0.1%	0.2%	0.2%	0.1%
**Step 4**					
Male	−0.253***	0.034	0.035	−0.018	−0.046
Married	−0.174**	−0.018	−0.086	0.076	−0.112
Risk group	0.044	−0.039	−0.024	−0.007	−0.023
Financial risk	0.134	0.152**	0.049	0.028	0.075
Previous anxiety		0.475***	0.348***	0.406***	0.543***
SOC	−0.526***	−0.317***	−0.348***	−0.370***	−0.268***
Support	−0.013	0.022	−0.039	−0.017	−0.055
SONC	0.010	0.017	−0.057	−0.057	0.027
*R* ^2^	39.3%***	50.2%***	43.1%***	50.6%***	53.7%***
ΔR^2^	0.0%	0.0%	0.3%	0.2%	0.1%

**p < 0.01, ***p < 0.001.

*Male – 1 = male, 0 = female; Married – 1 = married, 0 = not married; Risk group – 1 = yes, 0 = no; SOC, sense of coherence; Support, perceived social support; SONC, sense of national coherence.*

In the first phase, when we did not have previous anxiety in the regression, the first and the second steps were significant. In the first step male and financial risk were significant. In the second step SOC, married, and male predicted anxiety. Perceived social support and SONC that were entered in the third and the fourth steps, respectively, were not significant at all, but SOC, married, and male were still significant. Thus, the stronger the previous anxiety and the less the SOC, the higher levels of anxiety they reported each time they completed the questionnaire. Additionally, when the previous anxiety was not in the regression (in phase 1), female were more anxious than male, married people were more anxious than unmarried people, and the lower the level of SOC, the higher the levels of anxiety. Lastly, in phase 1 and 2, the higher the financial risk, the higher were the levels of anxiety. The findings confirmed the patterns suggested between the variables in the first hypothesis (1a,b,d) and confirmed the main role of SOC as predicting both mental health and anxiety (1c).

### Mean Differences in Mental Health, Anxiety, Coping Resources, and Risk Factor, Between the Research Phases

To test the second hypothesis whether there were differences in each of the main variables between the phases when the participants completed the questionnaires, one-way ANOVA for repeated measures were conducted, for each variable, separately. *Post hoc* Bonferroni tests were conducted in order to find the differences between times.

The pattern confirmed hypothesis 2a, related to the significantly decreased levels of anxiety from the first acute phase to the next four phases (see Table 5).

**TABLE 5:** Means and SD for each variable of the research, separately for each phase (*n* = 198).

Variables		Phase 1	Phase 2	Phase 3	Phase 4	Phase 5	*F*	η ^2^
Mental health	M	3.93	3.79	3.86	3.88	3.79		
	SD	1.05	0.98	0.97	0.97	1.01	*F*(4,788) = 2.47	0.012
Anxiety	M	1.03	0.74	0.83	0.79	0.75		
	SD	0.83	0.68	0.73	0.69	0.64	*F*(4,788) = 12.37***	0.059
SOC	M	4.61	4.61	4.59	4.62	4.65		
	SD	0.84	0.86	0.87	0.85	0.88	*F*(4,788) = 0.55	0.003
Support	M	3.42	3.16	3.15	3.03	3.01		
	SD	0.92	0.88	0.84	0.84	0.88	*F*(4,788) = 18.65***	0.086
SONC	M	4.17	4.20	3.74	3.83	3.82		
	SD	1.16	1.14	1.17	1.24	1.26	*F*(4,788) = 26.91***	0.120
Financial risk^+^	M	3.59	2.84	2.87	2.67	2.53		
	SD	1.23	1.26	1.14	1.15	1.14	*F*(4,716) = 52.20***	0.226

***p < 0.001.

*SOC, sense of coherence; Support, perceived social support; SONC, sense of national coherence.*

*^+^180 participants answered the question about financial risk as opposed to the other variables that were competed by all the 198 participants.*

However, unexpectedly, mental health levels did not significantly change between the remaining research phases (the decrease found was not significant). Therefore, hypothesis 2b was not confirmed.

As for the decrease in the coping resources of perceived social support and SONC, and increase in financial risk, the results confirmed the hypotheses (hypotheses 2c,d): The level of financial risk increased after the first phase and again after the third phase and levels of coping resources of perceived social support and SONC significantly decreased. Perceived social support levels decreased from the first phase to the next four phases of data collection. It continued to decrease again after the third phase to the last two phases. SONC also decreased after the second phase.

As expected, (hypothesis 2e) levels of SOC remained stable during the different phases of data collection (The analyses were conducted using demographic variables as covariate variable, and the results were the same).

## Discussion

The COVID-19 crisis shook the well-known reality and required populations all over the world to adjust to a new chaotic reality (Žižek, 2020). Employing the salutogenic approach (Antonovsky, 1979) we asked “Why, when people are exposed to the same stress which causes some to become ill, do some remain healthy?” (Antonovsky, 1979, p. 56). Our first research question related to the role of individual [sense of coherence (SOC)], social (perceived social support), and national [sense of national coherence (SONC)] coping resources, as well as situational and demographic factors (level of health and financial risks and gender), in predicting salutogenic outcomes (mental health) and also pathogenic outcomes (general anxiety) at different phases during the COVID-19 crisis.

The longitudinal study enabled us to examine the salutogenic hypotheses regarding the core concept of the model – the sense of coherence (Antonovsky, 1979). SOC was indeed found as the main predictor of both mental health and anxiety during all 5 phases of the pandemic. Moreover, people who had a stronger SOC at the beginning of the pandemic have been found to have a higher level of mental health after an extremely challenging year of COVID-19. SOC remained stable along the five phases of data collection while all the other coping resources significantly decreased.

These findings support Antonovsky’s (1979, 1987) assumption that the individuals’ ability to perceive the world as comprehensible, manageable, and meaningful, is a main and core coping resource among adults. SOC helps to identify and mobilize relevant resources to cope with stressors and manage tension successfully and, as a result of this process, it preserves health and well-being. Previous studies have confirmed the important role of SOC in predicting health in different kinds of crises (for review, see Eriksson and Mittelmark, 2017) and in the beginning of the COVID-19 pandemic (Schäfer et al., 2020; Mana et al., 2021a,b). This study, however, deepened our insight regarding the potential longitudinal effects of the individual resource SOC in the unique global context of the pandemic actually having transformed the whole world to an unpredictable and chaotic place for a very long time (Žižek, 2020). The pattern of sharp fluctuation of the pandemic waves challenged the process of adapting on both individual and group levels. Individuals and groups had to find new ways to adjust to the changing reality, to regain a sense of comprehensibility, and to continue in finding new meanings over the changing phases. This challenge has become more difficult as solutions of yesterday turned into the problems of today. The leaders’ crisis-management decisions (like lockdown) increased financial and mental health risk (Prati and Mancini, 2021) and currently even the main source of coping strategy relief (the vaccine) has not stopped the rise of another wave of a variant of COVID-19. However, according to our findings, these changing characteristics of the crisis have not changed the main salutogenic answer about the core individual resource for coping, the SOC.

Our findings also highlight the differences between the individual and national salutogenic resources: SOC and SONC. While both constructs contribute to mental health, the individual perception of the world as coherent was found to be a stable resource and the main coping asset, while the perception of ones’ nation as a source for comprehensibility, manageability, and meaningfulness decreased as the crisis continued. This finding reflects the impact of local processes which occurred during the COVID-19 pandemic, when the national collective and its leaders in many places around the world, including in Israel, failed to supply comprehensible regulations and explanations, a trustworthy and consistent way to control the crisis, or develop collective narratives that could give meaning to this difficult period (Louwerse et al., 2021). Thus, despite the importance that national resources might provide in collective crises (Levine and Scotch, 1970) our findings revealed that, at least in the case of COVID-19 in Israel, the nation did not succeed to serve as a stable resource. This might be explained partly by the political crisis in the country during this period (Mana and Sagy, 2020). Additional studies, however, are needed to explore this pattern in other countries.

Another contribution of the current study is the illumination of the different patterns of coping resources and situational factors involved in predicting a salutogenic outcome (mental health) and pathogenic outcome (general anxiety) in acute vs. chronic phases. While SOC, as mentioned above, was found as the main predictor of both mental health and anxiety at each of the research phases, the other factors had different roles in predicting mental health vs. anxiety levels (hypothesis 1), and in the process of evolving from acute to chronic phase (hypothesis 2) over time.

As expected, the salutogenic factor of mental health was predicted by social and national coping resources (perceived social support and SONC), while anxiety, as a pathogenic factor, was predicted by situational factors (level of risk and gender). These findings could reflect the situational characteristic of the anxiety measure versus the more habitual regular orientation in life of the mental health measure (Sagy, 2002). It seems that in understanding the psychological outcomes of crisis it is important not to limit the perspective only to salutogenic or pathogenic outcomes, but to integrate salutogenic and pathogenic approaches and measurements. This idea supports Antonovsky’s (1987) perspective that salutogenesis outcomes are not the opposite of pathogenesis outcomes, but rather stand alongside pathogenesis in a continuum from ease to dis-ease.

The results also confirmed the expected pattern of moving from acute to chronic stressful situation: levels of general anxiety were higher in the first phase of the pandemic outbreak as compared to the other phases. Levels of social and national coping resources significantly decreased over time and levels of financial risk increased. Mental health levels gradually decreased, but this decrease, unexpectedly, was not significant. It seems that while in the acute situation of the pandemic outbreak the overwhelming change and chaos in daily life resulted in a sharp and high arousal of anxious response that gradually decreased (Kudielka and Wüst, 2010), living in a continuing chronic stressful situation leads to a gradual decrease in coping resources (Lazarus and Folkman, 1984). Our findings support also the claim that it is difficult to determine a specific point when the acute stressful event became chronic, although acute and chronic stressful situations are different in nature (Gottlieb, 1997). However, the findings can indicate a cumulative stressful process during the first year of the pandemic. Therefore, it seems more appropriate to relate to the phases of acute and chronic stress as a process along a continuum. This finding has a meaningful warning aspect: it appears that as long as the crisis continues people have to struggle with the coming waves with less and less social and national coping resources.

Before concluding, several methodological limitations should be noted. First, choosing the phases of data collection was based on our attempt to explore periods of main changes in the pandemic waves. Since the nature of the crisis was complex, we did not want to consider one criterion only (for example, number of COVID-19 cases) but also other indicators like level of restrictions of the regulations. It seems that we succeeded to “catch” the main points, but, since we did not know in advance how the situation will be developed, we sometimes missed the waves’ peaks. Another limitation is the non-probability nature of the sampling and the attrition of participants between the phases of data collection.

To conclude, it seems that studying the longitudinal struggle with the COVID-19 pandemic could give us insights into the dynamic process of the development of an acute stressful situation into a chronic one. Although we employed the salutogenic approach, our study suggests that both salutogenic and pathogenic reactions are significant as indicators, each of them has a unique pattern of coping resources and risk factors as explanatory factors. Our main findings, however, indicate that in a global crisis, in which health and economic threats are experienced for a long period, the ability of the individual to perceive the world as comprehensible, manageable, and meaningful, has the most significant importance in coping.

In conclusion, our research findings may encourage researchers, health promoters, psychologists, and educational experts to seek ways to explore and develop strategies that can help foster increase in SOC, especially among children and adolescents as a preventative factor (Sagy, 2014) and salutogenic resource. In addition, understanding the importance of comprehensibility, manageability, and meaningfulness for the population in times of global crisis, can provide guidance to leaders in managing the crisis and formulating messages in order to increase the populations’ salutogenesis instead of provoking chaos.

## Data Availability Statement

The raw data supporting the conclusions of this article will be made available by the authors, without undue reservation.

## Ethics Statement

The studies involving human participants were reviewed and approved by Ethics Committee of the Ben-Gurion University of the Negev, Israel. The patients/participants provided their written informed consent to participate in this study.

## Author Contributions

AM devised the project, developed the theory, designed the study, directed the data collection in Israel, and took the lead in writing the manuscript with SS. OC, MN, and YM designed the computational framework, analyzed the data, and contributed to the writing of the manuscript. SB contributed to the writing of the manuscript. SS devised the international project, developed the theory, designed the study, directed the data collection in Israel, and took the lead in writing the manuscript with AM. All authors contributed to the manuscript and research.

## Conflict of Interest

The authors declare that the research was conducted in the absence of any commercial or financial relationships that could be construed as a potential conflict of interest.

## Publisher’s Note

All claims expressed in this article are solely those of the authors and do not necessarily represent those of their affiliated organizations, or those of the publisher, the editors and the reviewers. Any product that may be evaluated in this article, or claim that may be made by its manufacturer, is not guaranteed or endorsed by the publisher.

## Footnotes

1
ourworldindata.org
